# Influence of Genetic Variants on Disease Regression and Outcomes in HCV-Related Advanced Chronic Liver Disease after SVR

**DOI:** 10.3390/jpm11040281

**Published:** 2021-04-07

**Authors:** Georg Semmler, Teresa Binter, Karin Kozbial, Philipp Schwabl, David Chromy, David Bauer, Benedikt Simbrunner, Theresa Müllner-Bucsics, Bernhard Scheiner, Albert Stättermayer, Matthias Pinter, Petra Steindl-Munda, Michael Trauner, Peter Ferenci, Thomas Reiberger, Mattias Mandorfer

**Affiliations:** 1 Division of Gastroenterology and Hepatology, Department of Internal Medicine III, Medical University of Vienna, 1090 Vienna, Austria; georg.semmler@meduniwien.ac.at (G.S.); teresa.binter@meduniwien.ac.at (T.B.); karin.kozbial@meduniwien.ac.at (K.K.); philipp.schwabl@meduniwien.ac.at (P.S.); david.chromy@meduniwien.ac.at (D.C.); david.bauer@meduniwien.ac.at (D.B.); benedikt.simbrunner@meduniwien.ac.at (B.S.); theresa.bucsics@meduniwien.ac.at (T.M.-B.); bernhard.scheiner@meduniwien.ac.at (B.S.); albertfriedrich.staettermayer@meduniwien.ac.at (A.S.); matthias.pinter@meduniwien.ac.at (M.P.); petra.munda@meduniwien.ac.at (P.S.-M.); michael.trauner@meduniwien.ac.at (M.T.); peter.ferenci@meduniwien.ac.at (P.F.); mattias.mandorfer@meduniwien.ac.at (M.M.); 2 Vienna Hepatic Hemodynamic Lab, Medical University of Vienna, 1090 Vienna, Austria

**Keywords:** hepatitis C, PNPLA3, TM6SF2, MBOAT7, HSD17B13

## Abstract

Genetic variants including *PNPLA3-rs738409 C>G, TM6SF2-rs58542926 C>T, MBOAT7-rs641738 C>T,* and *HSD17B13-rs72613567 T>TA* have been shown to influence progression to advanced chronic liver disease (ACLD) in patients with chronic hepatitis C (CHC). We aimed to investigate their impact on disease regression (i.e., changes in hepatic venous pressure gradient [HVPG] and non-invasive surrogates [liver stiffness measurement (LSM), von Willebrand factor (VWF), and VWF/platelet count ratio (VITRO)]) and clinical outcomes after CHC cure in 346 patients with pre-treatment ACLD. Patients carrying the *PNPLA3* minor allele had more advanced liver disease prior to antiviral therapy, confirming its impact on liver disease progression. In a subgroup of 88 patients who underwent paired HVPG-measurements and were genotyped for all SNP/indels, *PNPLA3/TM6SF2/MBOAT7/HSD17B13* genotypes were not associated with changes in HVPG. In line, changes in non-invasive surrogates of portal hypertension (LSM/VWF/VITRO) were comparable between carriers and non-carriers of the *PNPLA3 G*-allele in the overall cohort. Finally, carriage of *PNPLA3 G*-allele was not associated with the development of hepatic decompensation, de-novo hepatocellular carcinoma, or transplant-free mortality during a median follow-up of 42 months after the end of antiviral treatment. Therefore, genetic variants in *PNPLA3/TM6SF2/MBOAT7/HSD17B13* do not impact the regression of portal hypertension and clinical outcomes in patients with pre-treatment ACLD after CHC cure.

## 1. Introduction

Interferon (IFN)-free therapies have revolutionized the treatment of patients with chronic hepatitis C (CHC). Rates of sustained virologic response (SVR, i.e., cure from hepatitis c virus (HCV infection) have increased to almost 100%, even in previous difficult-to-cure patient populations, such as patients with HIV coinfection [1,2] or compensated advanced chronic liver disease (cACLD) and portal hypertension [3,4]. Accordingly, the focus of attention has shifted to the regression of liver disease and personalized risk stratification.

After approximately 5 years of follow-up (FU), HCV cure by IFN-based therapies improved the histological stage of fibrosis in 82% of patients and in 61% despite previously established cirrhosis [5,6]. Similarly, portal hypertension is ameliorated in the majority of patients who achieved SVR [7,8,9,10]. Early decreases in HVPG may be primarily driven by a decrease in the dynamic component of increased intrahepatic resistance due to the resolution of inflammation and decrease in hepatic vascular tone (accounting for approximately 30% of intrahepatic resistance), while long-term changes may be a consequence of an improvement in the structural component (e.g., liver fibrosis) [11]. Nevertheless, a considerable proportion of patients may have reached a “point-of-no-return”—for instance, in a study of Mauro and colleagues [12] in patients with recurrent CHC after liver transplantation (LT), none of the patients with Lannaec stage 4C (i.e., severe cirrhosis in which at least one very broad septum or many micronodules) resolved cirrhosis.

Nowadays, liver biopsy—and, to some extent, hepatic venous pressure gradient (HVPG) measurement—have been replaced by non-invasive surrogate markers such as liver stiffness measurement (LSM), von Willebrand factor (VWF) antigen, and von Willebrand factor/platelet count ratio (VITRO) score [13,14]. Although an improvement of these prognostically relevant non-invasive tests has been reported [8], factors determining these changes are still under investigation.

A series of single nucleotide polymorphisms (SNP) and indels have been shown to modify the severity of CHC. The rs738409 G-allele encoding the I148M variant of patatin-like phospholipase domain-containing protein 3 (PNPLA3) [15] has been linked to hepatic steatosis [16,17,18] and liver fibrosis [16,18,19] in patients with CHC. Moreover, it impacts the outcome of patients with advanced chronic liver disease (ACLD) [20]. Further genome-wide association studies linked transmembrane 6 superfamily 2 (TM6SF2)-rs58542926 and membrane-bound O-acyltransferase domain-containing protein 7 (MBOAT7)-rs641738 variants with liver fibrosis in patients with CHC [21,22]. Finally, the rs72613567 TA-allele of the 17β-hydroxysteroid dehydrogenase type 13 (HSD17B13) gene has been associated with reduced risks of severe liver fibrosis in CHC [23], while it did not protect patients who have already progressed to ACLD from developing clinical events [24].

Although the impact of these genetic variants on liver disease progression is well-defined, their impact on liver disease regression after cure of CHC (i.e., after the removal of the primary etiologic factor) has yet to be investigated.

Therefore, we aimed to assess their impact on the regression of portal hypertension, as assessed by changes in (I) hepatic venous pressure gradient (HVPG) and (II) non-invasive markers (LSM, VWF, VITRO score) as well as (III) clinical events in CHC patients with ACLD who achieved SVR to IFN-free therapies.

## 2. Materials and Methods

### 2.1. Study Population and Design

**Figure 1:**
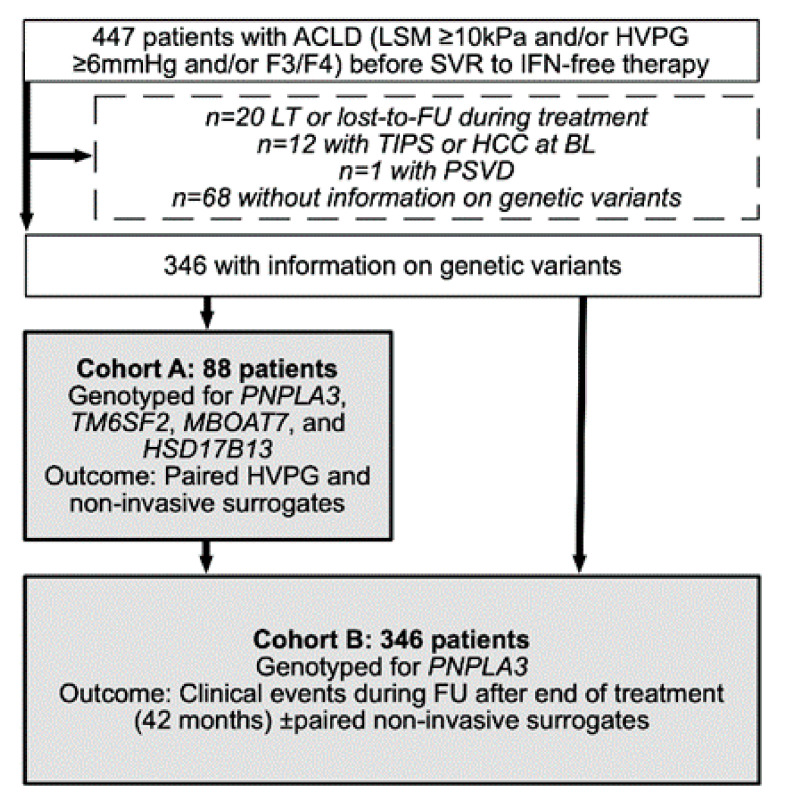
Study flow chart. Abbreviations: ACLD advanced chronic liver disease, FU follow-up, HCC hepatocellular carcinoma, HVPG hepatic venous pressure gradient, IFN interferon, LT liver transplantation, PSVD porto-sinusoidal vascular disease, SVR sustained virologic response, TIPS transjugular intrahepatic portosystemic shunt.

All patients who underwent IFN-free treatment at the Medical University of Vienna between 1 January 2014 and 31 July 2019 and achieved SVR were assessed for eligibility. The study was approved by the ethics committee of the Medical University of Vienna (EK: 1947/2019). Inclusion criteria were: (I) evidence suggestive or confirmative of ACLD (defined by baseline [BL] LSM ≥ 10 kPa, HVPG ≥ 6 mmHg, or advanced liver fibrosis/cirrhosis on histology [25]) and (II) data on genetic variants. Patients were excluded if they had undergone LT before the end of IFN-free treatment or if no clinical FU was available. Moreover, patients in whom a transjugular intrahepatic portosystemic shunt (TIPS) had been placed or who had been diagnosed with hepatocellular carcinoma (HCC) or porto-sinusoidal vascular disease (PSVD) [26] before the end of treatment were excluded. Finally, 346 patients were included (Figure 1).

Specifically, data on all investigated genetic variants (PNPLA3 rs738409 C>G, TM6SF2 rs58542926 C>T, MBOAT7 rs641738 C>T, and HSD17B13 rs72613567 T>TA) were available in 88 thoroughly characterized patients with paired measurements of HVPG as well as LSM and VITRO score (cohort A). Of note, these patients have been previously reported in a study investigating the prognostic value of changes in HVPG [8] as well non-invasive markers [14] after HCV eradication. Importantly, these studies did not provide information on genetic variants and their impact on liver disease regression.

In addition, we evaluated the influence of PNPLA3 rs738409 C>G (i.e., the common genetic variant that showed the strongest effect on liver disease progression in previous studies) on liver disease regression and post-treatment outcomes in the overall study population of 346 patients (cohort B).

### 2.2. Genotyping and Assessment of Linkage Disequilibrium

DNA was extracted using the QIAamp DNA Blood Mini Kit (QIAGEN, Germany). Genotyping was performed by StepOnePlus Real-Time PCR System and a TaqMan SNP Genotyping Assay (Applied Biosystems, USA). Linkage disequilibrium was investigated using LDmatrix (National Cancer Institute, https://ldlink.nci.nih.gov/?tab=ldmatrix, accessed on 1 April 2021).

### 2.3. HVPG Measurement (Cohort A)

HVPG measurements were performed by the Vienna Hepatic Hemodynamic Lab at the Medical University of Vienna following a standardized operating procedure [27] and in the absence of non-selective betablockers (NSBB) and nitrates. In patients on NSBB, treatment was interrupted 5 days before HVPG measurements. HVPG values ≥ 10 mmHg denoted clinically significant portal hypertension (CSPH) [25,28]. In patients with CSPH at BL, HVPG response to etiological therapy was defined by an HVPG decrease ≥ 10% after HCV eradication [7,8,25]. Response assessments were performed a median of 18.1 (31.3) weeks after the end of therapy.

### 2.4. Clinical and Laboratory Parameters (Cohorts A and B)

Clinical and laboratory parameters were evaluated by chart review. LSM and VITRO were assessed prior to antiviral therapy, as well as a median of 12.9 (24.1) weeks after the end of therapy (i.e., approximately at the time of SVR assessment). LSM was performed by vibration-controlled transient elastography (FibroScan^®^; Echosens, Paris, France) in fasting condition. Reliability of liver stiffness measurements was defined by previously established criteria [29]. Controlled attenuation parameter (CAP) was concomitantly assessed using the same device. Plasma VWF antigen levels were measured by a latex agglutination assay (STA LIATEST VWF, Diagnostica Stago, Asnieres, France). VITRO score was calculated by dividing plasma VWF antigen levels (%) by platelet count (PLT; G × L^−1^) [13].

### 2.5. Clinical Events during FU (Cohort B)

FU started at end of treatment. In patients with cACLD, first hepatic decompensation was defined by variceal bleeding, incident ascites, or incident hepatic encephalopathy (HE), whereas in patients with decompensated ACLD, further hepatic decompensation was defined by variceal bleeding, requirement of paracentesis, admission for grade 3/4 HE, or development of grade 3/4 HE during admission. Furthermore, de-novo HCC, TIPS placement, LT, and death were recorded.

### 2.6. HCV Therapy, Statistical Analyses and Ethics

See Supplementary Methods.

## 3. Results

### 3.1. Patient Characteristics

In 88 patients with data on all four SNP/indels (cohort A), 73 patients (83.0%) exhibited TM6SF2 rs58542926 C/C and 15 (17.0%) the C/T genotype (Table S1). Moreover, MBOAT7 rs641738 C/C genotype was present in 19 patients (21.6%), C/T in 49 (55.7%), and T/T in 20 (22.7%). Finally, 52 patients (59.1%) carried the HSD17B13 rs72613567 T/T genotype, while 31 (35.2%) and five (5.7%) were T/TA and TA/TA, respectively (in comparison to Hardy–Weinberg equilibrium for TM6SF2 rs58542926: Χ² = 0.764, MBOAT7 rs641738: Χ² = 1.139, and HSD17B13 rs72613567: Χ² = 0.018). There was no association between four investigated variants (Table S2).

Of 346 patients included in the overall cohort (cohort B), 173 patients (50.0%) carried the PNPLA3 rs738409 C/C genotype (i.e., wild type [WT]), 146 patients (42.2%) had the G/C genotype, and 27 patients (7.8%) had the G/G genotype (Hardy–Weinberg equilibrium: Χ² = 0.248). This cohort included 230 (66.5%) males and 116 (33.5%) females, with a mean age of 55.4 ± 10.7 years (Table 1). Forty-four patients (12.7%) had previous hepatic decompensation, and 92 patients (26.6%) had any esophageal/gastric varices. Mean BL-model for end-stage liver disease (MELD) score was 8.9 ± 2.8 points, median BL-LSM 17.7 (IQR: 11.8–28.5) kPa, mean BL-PLT 138 ± 65 G × L^−1^, median BL-VWF 239 (IQR: 173–321)%, and median BL-VITRO 1.91 (IQR: 1.10–3.38). One hundred and eighty-one patients (52.3%) had clinical evidence of CSPH (defined by history of hepatic decompensation, presence of varices, or BL-LSM ≥ 20 kPa).

When comparing pre-treatment characteristics of patients in the overall cohort (cohort B) carrying the PNPLA3 rs738409 G-allele to C/C patients, we found a higher prevalence of varices (32.4 vs. 20.8%, *p* = 0.015), a higher BL-MELD score (9.3 ± 3.1 vs. 8.4 ± 2.4 points, *p* = 0.001), more patients with evidence of CSPH (60.7 vs. 43.9%, *p* = 0.002), and a higher BL-VITRO score (2.05 [IQR:1.18–3.87] vs. 1.65 [IQR:0.99–3.23], *p* = 0.033; Table 1) in G-allele carriers. Of note, neither components of the metabolic syndrome or hepatic steatosis nor alcohol consumption or statin/NSBB use were significantly different between G-allele carriers and non-carriers, although the prevalence of metabolic comorbidities and especially hepatic steatosis (52.3%) was high (Table S3).

Analysing PNPLA3 rs738409 G/C and G/G genotypes separately, G/G patients displayed the highest BL-LSM (29.1 [IQR: 21.6–38.5] vs. 18.0 [IQR: 11.7–30.0] vs. 16.9 [IQR: 11.8–26.6] kPa, *p* = 0.005), and BL-VITRO (2.74 [IQR: 1.34–4.78] vs. 1.94 [IQR: 1.17–3.30] vs. 1.65 [IQR: 0.99–3.23], *p* = 0.038). Moreover, 81.5% of G/G patients had evidence of CSPH compared to 56.8 and 43.9% with G/C and C/C genotype, respectively (*p* < 0.001; Table S4).

### 3.2. Genetic Variants and Changes in HVPG (Cohort A)

Of note, there was no correlation between changes in HVPG and the time from treatment initiation (Spearman’s *ρ* = 0.097, *p* = 0.370) and end of treatment (*ρ* = 0.055, *p* = 0.609) to the second HVPG-assessment, and thus, the time point of the second HVPG measurement did not impact our results.

To investigate the impact of genetic variants on the regression of portal hypertension as assessed by HVPG, we compared the prevalence of genotypes for the abovementioned SNP/indels between patients with a decrease in HVPG and those without and found comparable frequencies (Table S1). Since HVPG response confers a clinical benefit in patients with pre-treatment CSPH [8], we compared patients with a ≥10% decrease in HVPG to those without and observed no differences in the distribution of SNP/indels (Table S5).

### 3.3. Genetic Variants and Changes in Non-Invasive Surrogates of Portal Hypertension (Cohort B)

Changes in non-invasive surrogates and the time from treatment initiation and end of treatment to the second HVPG assessment were not statistically significantly correlated (data not shown).

We compared the dynamics of surrogates in portal hypertension (PLT, LSM, VWF, and VITRO score) between patients with or without the PNPLA3 rs738409 G-allele in the overall study population (Table 2). Carriers of the G-allele showed lower BL- (130 ± 60 vs. 145 ± 66 G × L^−1^, *p* = 0.045) and FU-PLT (143 ± 74 vs. 160 ± 72 G × L^−1^, *p* = 0.031). Moreover, PNPLA3 G-allele carriers had higher BL- (2.06 [IQR: 1.19–4.00] vs. 1.59 [IQR: 0.97–3.22], *p* = 0.015) and FU-VITRO scores (1.64 [IQR: 0.79–2.71] vs. 1.12 [IQR: 0.67–2.17], *p* = 0.012). Importantly, there were no differences in the absolute or relative changes in non-invasive surrogates of portal hypertension between G-allele carriers and non-carriers. Noteworthy, this finding was not affected by accounting for BL values of the respective parameters by an analysis of covariance (ANCOVA).

Additionally, prevalence of hepatic steatosis declined in PNPLA3 rs738409 G-allele carriers (47.3 vs. 41.8%) and non-carriers (57.8 vs. 47.8%). Although BL and FU CAP values were not significantly different between carriers and non-carriers of the PNPLA3 rs738409 G-allele, absolute and relative changes showed a trend towards a discordancy (absolute Δ CAP: G-allele carriers: 4 (IQR: 59) vs. non-carriers: -6 (IQR: 64) dB × m^−1^, *p* = 0.087; relative Δ CAP: 1.5 (IQR: 27.0) % vs. -1.9 (IQR: 25.1) %, *p* = 0.096). Interestingly, absolute changes in CAP were significantly different between carriers and non-carriers of the PNPLA3 G-allele after adjusting for BMI in ANCOVA (*p* = 0.046).

### 3.4. Hepatic Decompensation during Follow-Up (Cohort B)

Next, we investigated the development of clinical events during FU. During a median FU of 42 (IQR: 22–54) months after the end of treatment, 23 patients (6.6%) developed hepatic decompensation. Specifically, nine patients (3.9%) showed development/worsening of ascites, eight patients (2.3%) had development/worsening of HE, and six patients (1.7%) suffered from variceal bleeding as first decompensation event post-treatment. Moreover, 25 patients (7.2%) developed HCC (corresponding to 2.4/100 patient years), one patient (0.3%) underwent TIPS placement, five patients (1.4%) underwent LT, and 20 patients (5.8%) died.

Differences in BL characteristics between patients with or without hepatic decompensation during FU included established indicators of hepatic dysfunction and portal hypertension such as a higher BL-MELD (12.1 ± 2.2 vs. 8.6 ± 2.7 points, *p* < 0.001), higher BL-LSM (39.3 [IQR: 28.0–59.1] vs. 17.2 [IQR: 11.8–27.0] kPa, *p* < 0.001), and higher BL-VITRO score (4.00 [IQR: 2.34–7.00] vs. 1.78 [IQR: 1.06–3.16], *p* < 0.001; Table S6).

Of note, the distribution of PNPLA3 genotypes was not significantly different between patients with and without hepatic decompensation during FU (*p* = 0.121).

**Figure 2:**
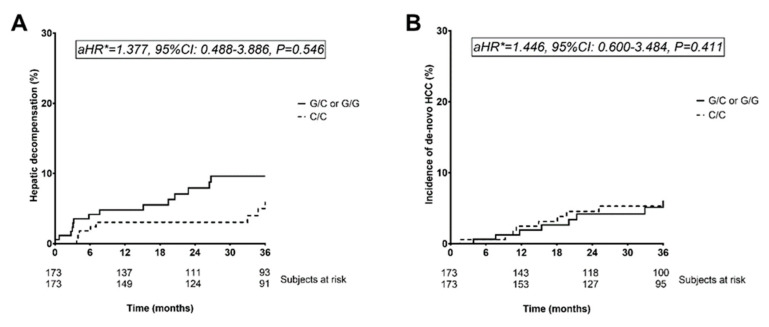
Kaplan–Meier analyses of (**A**) hepatic decompensation and (**B**) the incidence of de-novo HCC in the overall cohort (cohort B), comparing carriers and non-carriers of PNPLA3 rs738409 G-allele. * Adjusted hazard ratio (aHR) was calculated using Cox regression analysis adjusting for history of hepatic decompensation, baseline MELD score, and baseline albumin level. Abbreviations: aHR adjusted hazard ratio, HCC hepatocellular carcinoma, HR hazard ratio, PNPLA3 patatin-like phospholipase domain-containing protein.

In a multivariate Cox regression analysis investigating determinants of hepatic decompensation, carriage of the PNPLA3 rs738409 G-allele (adjusted hazard ratio [aHR]: 1.377, 95%CI: 0.488–3.886, *p* = 0.546) was not independently associated with hepatic decompensation, after correcting for important differences in BL characteristics, i.e., serum albumin level, MELD score, and history of hepatic decompensation (Table S7, Figure 2).

Moreover, subgroup analyses on first hepatic decompensation including only patients with cACLD (aHR: 3.685, 95%CI: 0.588–23.094, *p* = 0.164) and further decompensation in dACLD patients (aHR: 0.946, 95%CI: 0.247–3.628, *p* = 0.936) yielded no statistically significant differences (Figure S1).

### 3.5. De-Novo Hepatocellular Carcinoma Risk as Well as Transplant-Free and Liver-Related Mortality

The risk for de-novo HCC was comparable between carriers and non-carriers of the PNPLA3 rs738409 G-allele (aHR: 1.446, 95%CI: 0.600–3.484, *p* = 0.411; Figure 2). Finally, we compared the risks of transplant-free mortality as well as liver-related mortality between patients with and without the PNPLA3 minor allele (G/C or G/G). The risk of transplant-free mortality was similar (aHR: 0.746, 95%CI: 0.289–1.925, *p* = 0.545), as was liver-related transplant-free mortality (aHR: 1.089, 95%CI: 0.301–3.937, *p* = 0.897; Table S7, Figure S2).

## 4. Discussion

Although the genetic variants evaluated in this study have been shown to modulate liver disease progression, their impact on liver disease regression remained unclear. Interestingly, none of these genetic factors had an impact on the changes in HVPG in a thoroughly characterized cohort of 88 patients undergoing paired assessments. Moreover, the *PNPLA3 rs738409 G*-allele (i.e., a well-established genetic risk factor for liver disease progression—as confirmed by our study—and HCC development) did not impact changes in surrogates of portal hypertension (PLT, LSM, VWF, and VITRO) and the development of clinical events (i.e., hepatic decompensation, HCC, and transplant-free mortality/liver-related transplant-free mortality) after the removal of the primary etiologic factor in a cohort of 346 patients.

Due to the unprecedented advances in the treatment of CHC, the focus of attention has shifted to disease regression and risk stratification after SVR [30]. Furthermore, findings in patients who achieved HCV cure may also improve the understanding of the role of genetic factors after the removal of the primary etiologic factor in other CLD, e.g., NASH, which resolves in a substantial proportion of patients achieving weight loss [31] and for which approval of medical treatments is on the horizon [32]. In addition, our observations may also be extrapolated to ALD patients achieving abstinence, who are more difficult to study due to common fluctuations in alcohol consumption over time.

Firstly, we did not find conclusive evidence that *PNPLA3, TM6SF2, MBOAT7,* and *HSD17B13* genotypes impacted the regression of portal hypertension, as assessed by HVPG. Of note, HVPG measurement is not only the diagnostic gold standard for portal hypertension [27], but also confers important prognostic information in patients who achieved SVR [8].

Secondly, to mitigate the risk of type II error due to the limited number of patients undergoing paired invasive HVPG measurements, we evaluated the effect of the presumably most impactful variant (i.e., *PNPLA3*) on changes in well-validated surrogates of portal hypertension [13]. Importantly, these markers may confer prognostic information, even beyond their association with HVPG [33,34], and post-treatment LSM and VITRO have previously been shown to predict direct endpoints after SVR, such as the development of hepatic decompensation and HCC [14,35,36]. We observed a clear association between the *PNPLA3 rs738409 G*-allele carriage and pre-treatment severity of hepatic dysfunction and portal hypertension, which is in line with the previous literature [20]. Patients with the *PNPLA3 G*-allele were considerably younger, which points towards an accelerated progression to ACLD. However, despite the evidence supporting the impact of *PNPLA3 rs738409 G*-allele carriage on liver disease progression, changes in surrogates of portal hypertension were comparable between carriers and non-carriers of the *PNPLA3 rs738409 G*-allele, indicating the absence of an impact on liver disease regression.

Finally, carrying the *PNPLA3 rs738409 G*-allele did not increase the risks of hepatic decompensation, transplant-free mortality, and liver-related transplant-free mortality, which is in line with our findings regarding HVPG, and non-invasive surrogates, however, contrasts a previous report based on a small cohort of 56 patients [37]. In this study by Dunn and co-workers, patients carrying the *PNPLA3 rs738409 G*-allele did not have more severe liver disease at BL but showed less pronounced decreases in CTP score after HCV cure. However, this study only included CTP B/C patients who are usually decompensated and may have even crossed a point of no return in liver disease [7]. Interestingly, a recent study indicated that in this particular group of patients, treatment-induced improvements of indicators of hepatic function (e.g., MELD score) do not necessarily translate into improved outcomes, questioning the clinical significance of such an endpoint [38]. In contrast to the study by Dunn and co-workers [37], which exclusively included patients with (usually decompensated) CTP stage B/C cirrhosis, our study included all clinical stages of ACLD, although it primarily comprised cACLD patients. The selective inclusion of patients with advanced hepatic impairment (i.e., CTP stages B/C) in the study by Dunn and colleagues [37] may have also obscured a potential link between *PNPLA3* genotype and BL severity of liver disease (i.e., liver disease progression), since CTP A cirrhosis could have been more common in those without a *G*-allele, as observed in our cohort of unselected ACLD patients. In another study, patients who survived the first 90 days after severe alcoholic hepatitis and achieved abstinence had a worse outcome, if they were homozygous for the *PNPLA3 rs738409 G*-allele [39]. Several other studies found an allele dose-dependent effect of *PNPLA3 rs738409* [16,18,40]. However, in line with previous studies from our group [17,20,41], the *PNPLA3 rs738409 G/G* genotype was only prevalent in 27 patients (7.8%), which did not allow for a comprehensive statistical analysis when applying a recessive model.

Nevertheless, we found a trend towards a higher risk of hepatic decompensation in patients carrying the *PNPLA3 rs738409 G*-allele in univariate analysis. However, the increased risk was primarily due to more advanced liver disease at BL, as the HR decreased considerably, after adjusting for known differences in BL characteristics.

The incidence of HCC after HCV eradication was comparable across *PNPLA3 rs738409* genotypes. Importantly, studies reporting an increased incidence of HCC in CHC patients who are homozygous for the *PNPLA3 rs738409 G*-allele, suggesting a cancerogenic effect of this variant—possibly due to increases in hepatic inflammation [18]—were mostly performed prior to the availability of IFN-free therapies [42]. Thus, they were conducted in the absence of highly effective therapies for CHC in patients with ACLD, which limits the applicability of their findings to current cohorts in which HCV eradication is achieved in basically all patients within a short period of time. Nevertheless, HCC incidence after SVR was higher than the incidence of hepatic decompensation, which is in line with the literature [43] ranging between 1.5–3.7/100 patient years in ACLD patients [43,44,45].

Following the description of the role of *PNPLA3 rs738409 G*-allele in CLD [15], common genetic variants have been extensively studied, as they may facilitate risk stratification, reveal therapeutic targets, and promote personalized therapy for CLD. Specifically, SNP in *PNPLA3, TM6SF2, MBOAT7* have repeatedly been shown to increase the susceptibility for and promote poor outcomes in NAFLD and ALD, while the indel in *HSD17B13* assessed in our study was protective in previous analyses. Moreover, there is a growing body of evidence indicating that these genetic variants also impact disease severity in patients with CHC [16,17,18,19,21,22,23,40,46,47], although the findings were not always consistent [24,41]. Hepatic steatosis has been identified as a risk factor for liver fibrosis in patients with CHC [48,49], which may partly explain the harmful effect of these genetic variants during active HCV infection. A recent study in HIV/HCV coinfected patients by our group linked hepatic steatosis with persistent necro-inflammatory activity and less pronounced decreases in HVPG after HCV cure, which would suggest that metabolic factors impede the regression of liver disease [50]. However, this previous study did not provide information on the genetic traits that have been shown to impact steatosis and steatohepatitis. Interestingly, genetic variants did not influence liver disease regression in our study, although we observed a trend towards a discordancy in the evolution of CAP between carriers and non-carriers of the *PNPLA3 rs738409 G*-allele (i.e., decreases in non-carriers vs. increases in carriers). Nevertheless, the diagnostic ability of CAP in patients with ACLD is limited [51], and the impact of genetic variants on long-term outcomes has yet to be investigated.

Since our findings suggest that the negative impact of genetic factors predisposing for progressive liver disease may be overcome by etiological cure, patients with an unfavorable genetic background may be subjected to more aggressive etiological treatment strategies (i.e., personalized therapy). Although this observation has limited relevance in the context of CHC, which has become easy to cure and should be treated in basically all patients without delay [52] to achieve the elimination goals, it could be of relevance for patient counselling/motivation in other etiologies of CLD such as NASH or ALD.

The significance of our findings is limited by the short time frame in which the changes in HVPG and non-invasive surrogates of portal hypertension were evaluated, as previous studies reported even further changes in HVPG if reevaluated at later time points. Accordingly, our data on HVPG and non-invasive surrogates may have primarily captured decreases in the dynamic component of intrahepatic resistance. However, an assessment at a later time point may have increased the number of unevaluable patients and, thus, may have introduced a relevant selection bias. Nevertheless, studies evaluating these parameters at a later time point would be desirable. Moreover, not all patients were evaluated by paired HVPG measurements, and we abstained from including information on paired liver biopsies due to very limited numbers, although the latter may have been particularly informative. However, our cohort of 88 patients is still the second-largest reported in the literature so far, and the largest studies to date did not include genetic information [9,10]. In addition, patients in cohort B were not genotyped for all genetic variants; however, we have evaluated the presumably strongest genetic factor (i.e., *PNPLA3 rs738409*) in the overall cohort, which helped to reduce the probability of type I error due to the investigation of a high number of genetic variants. Although our study aimed to investigate the impact of genetic factors, metabolic factors and medical treatments may also determine clinical outcomes after HCV eradication. In this context, we ruled-out that components of the metabolic syndrome, hepatic steatosis, alcohol consumption, and statin—as well as NSBB use—had a major impact on our findings by demonstrating that all of these factors were well balanced between patients with or without the *PNPLA3 rs738409* minor allele. Finally, our study lacked a comparison group of CHC patients who did not achieve SVR; however, the impact of the genetic variants on disease progression has previously been established [16,18,19,47]. In order to account for the between-genotype difference in the severity of liver disease at BL, we applied three different approaches, depending on whether the impact of genetic factors on indicators of portal hypertension severity (e.g., HVPG, PLT, LSM, VWF, and VITRO score), or direct clinical endpoints were analyzed. First, for analyses on indicators of portal hypertension severity, we primarily relied on relative changes or stratified by relative decreases (i.e., HVPG response), thereby minimizing the impact of BL differences on the observed treatment-induced changes. Second, we have included an additional, possibly more robust statistical analysis to compare the evolution of non-invasive surrogates of portal hypertension (PLT, LSM, VWF, and VITRO score) in cohort B. This form of analysis (ANCOVA) also considered the BL values of the respective variables and thus, directly accounted for pre-treatment differences. Third, analyses on direct clinical endpoints were adjusted for hepatic decompensation, BL MELD score, and BL albumin levels in order to account for differences in BL characteristics. Following these approaches, we did not observe any differences between carriers and non-carriers of *PNPLA3 rs738409 G*-allele. However, due to a limited number of events, and thus a number of variables that can be included in multivariate models, we were unable to adjust the time-to-event analyses for all differences in BL characteristics. In addition, there may have been differences in unknown factors, that we were unable to account for. Accordingly, our findings clearly require confirmation by further studies.

## 5. Conclusions

In conclusion, genetic variants in *PNPLA3, TM6SF2, MBOAT7,* and *HSD17B13* do not seem to impact the short-term regression of portal hypertension. Moreover, *PNPLA3* genotype does not affect clinical outcomes in patients with pre-treatment ACLD within the first years after HCV cure. Future studies should investigate whether this also applies to long-term outcomes and other etiologies of CLD after the removal of the primary etiologic factor.

## Figures and Tables

**Table 1:** Patient characteristics in the overall cohort (cohort B) and comparison of patients with the PNPLA3 rs738409 G-allele, or without. Abbreviations: ALT alanine aminotransferase, AST aspartate aminotransferase, BL baseline, CSPH clinically significant portal hypertension, CTP Child–Turcotte–Pugh score, GGT gamma-glutamyltransferase, INR international normalized ratio, LSM liver stiffness measurement, MELD model for end-stage liver disease, PLT platelet count, PNPLA3 patatin-like phospholipase domain-containing protein 3, VITRO von Willebrand factor/platelet count ratio, VWF von Willebrand factor.

Patient Characteristics	All Patients, *n* = 346	*C/C*, *n* = 173	*G/C* or *G/G*, *n* = 173	*p* Value
Age, years	55.4 ± 10.7	57.2 ± 10.8	53.7 ± 10.4	0.002
Sex				
Male	230 (66.5%)	109 (63.0%)	121 (69.9%)	0.172
Female	116 (33.5%)	64 (37.0%)	52 (30.1%)	
History of hepatic decompensation	44 (12.7%)	18 (10.4%)	26 (15.0%)	0.197
Varices	92 (26.6%)	36 (20.8%)	56 (32.4%)	**0.015**
Small	46 (13.5%)	18 (10.4%)	29 (16.8%)	0.051
Large	43 (12.6%)	18 (10.4%)	27 (15.6%)	
BL-CTP score, points	5 ± 1	5 ± 1	6 ± 1	**0.015**
Stage A	304 (87.9%)	161 (93.1%)	143 (82.7%)	**0.003**
Stage B/C	42 (12.1%)	12 (6.9%)	30 (17.3%)	
BL-MELD score, points	8.9 ± 2.8	8.4 ± 2.4	9.3 ± 3.1	**0.001**
BL-LSM ^1^, kPa	17.7 (11.8–28.5)	16.9 (11.8–26.6)	20.9 (11.8–32.8)	0.098
Evidence of CSPH ^2^	181 (52.3%)	76 (43.9%)	105 (60.7%)	**0.002**
BL-PLT, G × L^−1^	138 ± 65	145 ± 66	130 ± 63	**0.045**
BL-VWF ^3^, %	239 (173–321)	232 (167–307)	249 (185–334)	0.127
BL-VITRO ^3^	1.91 (1.10–3.38)	1.65 (0.99–3.23)	2.05 (1.18–3.87)	**0.033**
BL-bilirubin, mg × dL^−1^	0.73 (0.54–1.13)	0.70 (0.55–0.98)	0.81 (0.53–1.23)	0.072
BL-creatinine, mg × dL^−1^	0.78 (0.68–0.91)	0.79 (0.70–0.92)	0.77 (0.67–0.91)	0.335
BL-albumin, g × L^−1^	40.6 ± 4.9	40.7 ± 4.9	40.4 ± 5.0	0.585
BL-INR	1.18 ± 0.27	1.13 ± 0.18	1.22 ± 0.33	**0.003**
BL-AST, U × L^−1^	67 (43–101)	64 (41–97)	71 (45–105)	0.260
BL-ALT, U × L^−1^	69 (42–107)	64 (43–105)	73 (38–109)	0.528
BL-GGT, U × L^−1^	93 (52–160)	89 (50–165)	94 (56–149)	0.800

^1^ Information available in 310 patients; ^2^ defined as history of hepatic decompensation, presence of varices, or LSM ≥ 20 kPaM; ^3^ information available in 328 patients. Bold values indicate statistical significance (*p* < 0.05).

**Table 2:** Comparison of dynamics in LSM, VWF, PLT, and VITRO score between patients of cohort B with the PNPLA3 rs738409 G-allele, or without. Abbreviations: BL baseline, FU follow-up, LSM liver stiffness measurement, PLT platelet count, VWF von Willebrand factor, VITRO von Willebrand factor antigen/platelet count ratio.

Patient Characteristics	*C/C*, *n* = 173	*G/C or G/G*, *n* = 173	*p* Value *	*p* Value **
BL-liver stiffness ^1^, kPa	16.8 (11.8–26.6)	20.9 (11.8–32.7)	0.129	-
Absolute Δ LSM ^1^, kPa	−3.4 (−7.4–(0.2))	−4.4 (-9.0-(−0.5))	0.620	0.720
Relative Δ LSM ^1^, %	−23.1 (−38.9–(−1.5))	−20.4 (−45.3–(−0.3))	0.738	0.885
FU-LSM^1^, kPa	13.5 (8.8–19.8)	14.2 (8.6–26.3)	0.491	0.720
BL-CAP^2^, dB × m^−1^	253 ± 60	242 ± 60	0.246	-
BL prevalence of hepatic steatosis ^2^, %	52 (57.8%)	43 (47.3%)	0.156	-
Absolute Δ CAP^2^, dB × m^−1^	−6 (64)	4 (59)	0.087	0.145
Relative Δ CAP ^2^, %	−1.9 (25.1)	1.5 (27.0)	0.096	0.312
FU-CAP^2^, dB × m^−1^	243 ± 52	246 ± 52	0.643	0.145
FU prevalence of hepatic steatosis ^2^, %	43 (47.8%)	38 (41.8%)	0.415	-
BL-PLT, G × L^−1^	145 ± 66	130 ± 63	**0.045**	-
Absolute Δ PLT, G × L^−1^	11 (−3–29)	6 (−5–20)	0.069	0.154
Relative Δ PLT, %	8.5 (−2.8–20.3)	5.8 (−5.5–17.7)	0.130	0.093
FU-PLT, G × L^−1^	160 ± 72	143 ± 74	**0.031**	0.154
BL-VWF ^3^, %	231 (167–304)	251 (185–339)	0.065	-
Absolute Δ VWF ^3^, %	−36 (−73–(-10))	−39 (−91–(11))	0.624	0.622
Relative Δ VWF ^3^, %	−18.3 (−30.3–(−5.1))	−17.8 (−31.0–(−5.7))	0.927	0.908
FU-VWF ^3^, %	178 (135–241)	193 (145–269)	0.231	0.622
BL-VITRO ^3^	1.59 (0.97–3.22)	2.06 (1.19–4.00)	**0.015**	-
Absolute Δ VITRO ^3^	−0.31 (−1.00–(−0.07))	−0.35 (−0.94–(0–12))	0.639	0.498
Relative Δ VITRO ^3^, %	−24.9 (−40.6–(−6.1))	−21.7 (−37.5–(6.5))	0.323	0.480
FU-VITRO ^3^	1.12 (0.67–2.17)	1.64 (0.79–2.71)	**0.012**	0.498

* Student’s *t*-test/Mann–Whitney U test or Chi-squared/Fisher’s exact test, as applicable; ** analysis of covariance; ^1^ information on paired measurements available in 299 patients; ^2^ defined by CAP ≥ 248 dB × m^−1^. Only reported for patients with paired measurements (available in 181 patients); ^3^ information on paired measurements available in 311 patients. Bold values indicate statistical significance (*p* < 0.05).

## Data Availability

The data are available from the authors at request.

## Supplementary Materials

The following are available online at https://www.mdpi.com/article/10.3390/jpm11040281/s1, Supplementary methods and results; Table S1. Baseline characteristics of cohort A and comparison between patients with a HVPG decrease or without, Table S2. Cross tables of genetic variants in cohort A, Table S3. Comparison of factors related to the metabolic syndrome, alcohol consumption, and NSBB use in the overall cohort (cohort B), and comparison of patients with the PNPLA3 rs738409 G-allele or without, Table S4. Patient characteristics in the overall cohort (cohort B) and comparison between PNPLA3 rs738409 genotypes, Table S5. Baseline characteristics of cohort A patients with CSPH and comparison between patients with a HVPG decrease ≥ 10%, or without, Table S6. Patient characteristics of the overall cohort (cohort B) and comparison between patients with hepatic decompensation during FU, or without, Table S7. Cox regression analyses on the influence of the PNPLA3 rs738409 G-allele (A) on hepatic decompensation and (B) on transplant-free mortality in the overall cohort (cohort B), Figure S1. Kaplan–Meier analyses on (A) first hepatic decompensation in patients with cACLD and (B) further decompensation in dACLD patients of the overall cohort (cohort B), comparing carriers and non-carriers of PNPLA3 rs738409 G-allele, Figure S2. Kaplan–Meier analyses on (A) transplant-free mortality and (B) liver-related mortality in the overall cohort (cohort B), comparing carriers and non-carriers of PNPLA3 rs738409 G-allele, Figure S3. Heatmap matrix of linkage disequilibrium statistics for TM6SF2 rs58542926 and MBOAT7 rs641738 based on the “Toscani in Italia” (TSI) cohort using LDmatrix.
