# Supplementary material for: Influence of Genetic Variants on Disease Regression and Outcomes in HCV-Related Advanced Chronic Liver Disease after SVR
Source: J Pers Med. 2021 Apr 7;11(4):281. doi: 10.3390/jpm11040281 (PMC8067986; doi:10.3390/jpm11040281)
Supplement: Supplementary file 1 [file jpm-11-00281-s001.zip › jpm-1142252-supplementary.pdf]

ELECTRONIC SUPPLEMENTARY MATERIAL

Influence of genetic variants on disease regression and outcomes in HCV-related advanced chronic liver disease after SVR

Georg SEMMLER[1, 2], M.D.

Teresa BINTER[1, 2], M.D.

Karin KOZBIAL[1], M.D.

Philipp SCHWABL[1, 2], M.D.

David CHROMY[1, 2], M.D.

David BAUER[1, 2], M.D.

Benedikt SIMBRUNNER[1, 2], M.D.

Theresa BUCSICS[1, 2], M.D.

Bernhard SCHEINER[1, 2], M.D.

Albert Friedrich STÄTTERMAYER[1, 2], M.D.

Matthias PINTER[1, 2], M.D. Ph.D.

Petra STEINDL-MUNDA[1], M.D.

Michael TRAUNER[1], M.D.

Peter FERENCI[1], M.D.

Thomas REIBERGER[1, 2], M.D.

Mattias MANDORFER[1, 2], M.D., Ph.D.

[1]Division of Gastroenterology and Hepatology, Department of Internal Medicine III, Medical University of Vienna, Vienna, Austria

[2]Vienna Hepatic Hemodynamic Lab, Medical University of Vienna, Vienna, Austria

G.S. and T.Bi. contributed equally to the manuscript.

Corresponding author: Thomas REIBERGER, M.D.

Division of Gastroenterology and Hepatology,

Department of Internal Medicine III,

Medical University of Vienna,

Währinger Gürtel 18-20,

1090, Vienna, Austria

Phone: +43 1 40400 47440

Fax: +43 1 40400 47350

E-Mail: thomas.reiberger@meduniwien.ac.at

SUPPLEMENTARY METHODS

HCV therapy

The choice of treatment regimen was at the physicians' discretion and depended on the availability of early-access programs, reimbursement policies, as well as national and international clinical practice guidelines at the time of treatment initiation. Treatment durations ranged from 8 to 24 weeks.

Statistical analyses

Statistical analyses were performed using IBM SPSS Statistics 25 (SPSS Inc., Armonk, New York, USA) and GraphPad Prism 8 (GraphPad Software, La Jolla, California, USA). Depending on their distribution, continuous variables were reported as mean ±standard deviation (SD) or median (IQR). Categorical variables were presented as numbers and proportions of patients. Comparisons of continuous variables were performed using Student's t-test or Mann-Whitney U test, as applicable. For comparisons of more than two groups, Kruskal-Wallis one-way analysis of variance was used. Proportions of patients were compared using Chi-squared test. The impact of the *PNPLA3 G-allele* on the evolution of non-invasive surrogates of portal hypertension (PLT, LSM, VWF, and VITRO score) in **cohort B** was additionally investigated by analysis of covariance (ANCOVA) adjusting for BL values of the respective parameters as well as BL BMI for controlled attenuation parameter (CAP) values. Spearman's rank correlation coefficients were calculated for assessing correlations.

Hardy-Weinberg equilibrium was used to calculate the difference between expected and observed genotype frequencies with X^2-values of ≥3.84 indicating sample ascertainment bias[1].

Time-to-event analyses were performed by the Kaplan-Meier method. Univariate and multivariate Cox regression analyses were conducted to determine factors associated with clinical endpoints. Patients were censored at the time of LT in all time-to-event analyses. A two-sided *P* value ≤0.05 was considered as statistically significant.

Ethics

This study was approved by the ethics committee of the Medical University of Vienna (EK: 1947/2019). Since this is a retrospective analysis, the requirement of a written informed consent was waived by the ethics committee.

SUPPLEMENTARY RESULTS

Assessment of linkage disequilibrium

Since *PNPLA3 rs738409* (chromosome 22) and *HSD17B13 rs72613567* (chromosome 4) are located on different chromosomes, we abstained from analyses for these variants. There was no evidence of a linkage disequilibrium between *TM6SF2 rs58542926 and MBOAT7 rs641738* (D'=0.0126, R^2=0.0001, X^2=0.0117, *P*=0.9138). (Supplementary Fig. 3).

Table S1

Patients characteristics	All patients,<br><br>n=88	HVPG-decrease,<br><br>n=67	No HVPG-decrease,<br><br>n=21	*P* value
Age, years	55.5±8.9	54.8±9.0	53.9±8.6	0.687
Sex				
Male	65 (73.9%)	48 (71.6%)	17 (81.0%)	0.397
Female	23 (26.1%)	19 (28.4%)	4 (19.0%)	
BMI, kg x m^{-2}	25.5±4.4	25.2±4.1	26.4±5.2	0.281
≥25 kg x m^{-2}	44 (50.0%)	33 (49.3%)	11 (52.4%)	0.803
≥30 kg x m^{-2}	13 (14.8%)	8 (11.9%)	5 (23.8%)	0.287
Alcohol consumption				
Abstinent	75 (85.2%)	58 (86.6%)	17 (81.0%)	0.121
Non-abstinent but below threshold[1]	8 (9.1%)	7 (10.4%)	1 (4.8%)	
Above threshold[1]	5 (5.7%)	2 (3.0%)	3 (14.3%)	
PNPLA3 rs738409				
C/C	41 (46.6%)	33 (49.3%)	8 (38.1%)	0.658
C/G	37 (42.0%)	27 (40.3%)	10 (47.6%)	
G/G	10 (11.4%)	7 (10.4%)	3 (14.3%)	
G/C or *G/G*	47 (53.4%)	34 (50.7%)	13 (61.9%)	0.371
TM6SF2 rs58542926				
C/C	73 (83.0%)	55 (82.1%)	18 (85.7%)	1.000
C/T	15 (17.0%)	12 (17.9%)	3 (14.3%)	
MBOAT7 rs641738				
C/C	19 (21.6%)	15 (22.4%)	4 (19.0%)	0.803
C/T	49 (55.7%)	36 (53.7%)	13 (61.9%)	
T/T	20 (22.7%)	16 (23.9%)	4 (19.0%)	
HSD17B13 rs72613567				
T/T	52 (59.1%)	40 (59.7%)	12 (57.1%)	0.940
T/TA	31 (35.2%)	23 (34.3%)	8 (38.1%)	
TA/TA	5 (5.7%)	4 (6.0%)	1 (4.8%)	
Allele score, points	1 (0-2)	1 (1-2)	2 (0-2)	0.766
History of hepatic decompensation	13 (15.5%)	9 (14.1%)	4 (20.0%)	0.498
Varices	33 (37.5%)	21 (31.3%)	12 (57.1%)	**0.033**
Small	16 (18.2%)	12 (17.9%)	4 (19.0%)	**0.034**
Large	17 (19.3%)	9 (13.4%)	8 (38.1%)	

BL-CTP score, points	5±1	5±1	6±1	0.250
Stage A	79 (89.8%)	60 (89.6%)	19 (90.5%)	1.000
Stage B/C	9 (10.2%)	7 (10.4%)	2 (9.5%)	
BL-MELD score, points	9.3±2.4	9.0±1.9	10.5±3.5	0.075
BL-HVPG, mmHg	13.9±5.4	13.7±5.2	14.5±5.9	0.527
6-9mmHg	23 (26.1%)	19 (28.4%)	4 (19.0%)	
10-15mmHg	29 (33.0%)	22 (32.8%)	7 (33.3%)	0.659
≥16mmHg	36 (40.9%)	26 (38.8%)	10 (47.6%)	
CSPH	65 (73.9%)	48 (71.6%)	17 (81.0%)	0.397
BL-LSM[2], kPa	22.0 (16.9-34.5)	21.3 (16.4-31.6)	28.6 (19.1-51.2)	0.070
BL-PLT, G x L^{-1}	108±50	113±49	93±49	0.109
BL-VWF[3], %	270 (194-349)	270 (204-332)	273 (160-373)	0.758
BL-VITRO[4]	2.83 (1.64-4.40)	2.82 (1.56-3.93)	3.57 (1.78-6.38)	0.166

[1] >30g/day and >20g/day for males and females, respectively[2].

[2] Information available in 85 patients.

[3] Information available in 86 patients.

Table S1. Baseline characteristics of **cohort A** and comparison between patients with a HVPG decrease, or without.

Abbreviations:

BMI body mass index

CSPH clinically significant portal hypertension

CTP Child-Turcotte-Pugh score

HSD17B13 hydroxysteroid 17-beta dehydrogenase 13

LSM liver stiffness measurement

MBOAT7 membrane bound O-acyltransferase domain containing 7

MELD model for end-stage liver disease

PLT platelet count

PNPLA3 patatin-like phospholipase domain-containing protein 3

TM6SF2 transmembrane 6 superfamily member 2

VITRO von Willebrand factor antigen/platelet count ratio

VWF von Willebrand factor

Table S2

		PNPLA3 rs738409		*P* value
		C/C	*C/G* or *G/G*	
TM6SF2 rs58542926	*C/C*	32	41	*0.253*
	C/T or *T/T*	9	6	
MBOAT7 rs641738	*C/C*	8	11	*0.658*
	C/T or *T/T*	33	36	
HSD17B13 rs72613567	*T/T*	21	31	*0.161*
	T/TA or *TA/TA*	20	16	
		TM6SF2 rs58542926		
		C/C	*C/T* or *T/T*	
MBOAT7 rs641738	*C/C*	14	5	*0.300*
	C/T or *T/T*	59	10	
HSD17B13 rs72613567	*T/T*	45	7	*0.283*
	T/TA or *TA/TA*	28	8	
		MBOAT7 rs641738		
		C/C	*C/T*	
HSD17B13 rs72613567	*T/T*	12	40	*0.684*
	T/TA or *TA/TA*	7	29	

Table S2. Cross tables of genetic variants in **cohort A**.

Abbreviations:

HSD17B13 hydroxysteroid 17-beta dehydrogenase 13

MBOAT7 membrane bound O-acyltransferase domain containing 7

PNPLA3 patatin-like phospholipase domain-containing protein 3

TM6SF2 transmembrane 6 superfamily member 2

Table S3

Patient characteristics	All patients, n=346	*C/C*, n=173	*G/C or G/G*, n=173	*P* value
BMI[1], kg x m^{-2}	27.0±5.0	27.3±5.1	26.6±4.9	0.258
≥25kg x m^{-2}	214 (62.4%)	108 (63.5%)	106 (61.3%)	0.666
≥30kg x m^{-2}	81 (23.6%)	42 (24.7%)	39 (22.5%)	0.637
Prediabetes[2]	36 (10.4%)	17 (9.8%)	19 (11.0%)	0.725
Diabetes[3]	58 (16.8%)	30 (17.3%)	28 (16.2%)	0.773
Arterial hypertension[4]	112 (32.4%)	63 (36.4%)	49 (28.3%)	0.108
Hypertriglyceridemia[5]	41 (11.8%)	20 (11.6%)	21 (12.1%)	0.868
HDL below threshold[6]	72 (20.8%)	36 (20.8%)	36 (20.8%)	1.000
Statin-use	15 (4.3%)	9 (5.2%)	6 (3.6%)	0.428
Hepatic steatosis[7]	112 (52.3%)	62 (55.4%)	50 (49.0%)	0.354
Alcohol consumption				
Abstinent	272 (78.6%)	141 (81.5%)	131 (75.7%)	0.423
Non-abstinent but below the threshold[8]	39 (11.3%)	17 (9.8%)	22 (12.7%)	
Above the threshold[9]	35 (10.1%)	15 (8.7%)	20 (11.6%)	
NSBB-use	89 (25.7%)	38 (22.0%)	51 (29.5%)	0.110

[1] Information available in 343 patients.

[2] Fasting blood glucose 100-125mg x dL^{-1}.

[3] Fasting blood glucose >125mg x dL^{-1}, HbA1c ≥6.5%, or antidiabetic medication.

[4] Blood pressure >140/90mmHg, or antihypertensive medication.

[5] Fasting triglyceride levels >150mg x dL^{-1}.

[6] <35mg x dL^{-1} for males and <39mg x dL^{-1} for females.

[7] Controlled attenuation parameter ≥248dB x m^{-1}. Data were available in 214 patients.

[8] >30g/day and >20g/day for males and females, respectively[2].

Table S3. Comparison of factors related to the metabolic syndrome[3], alcohol consumption, and NSBB-use in the overall cohort (**cohort B**), and comparison of patients with the *PNPLA3 rs738409 G*-allele, or without.

Abbreviations: BMI body mass index

NSBB non-selective betablockers

Table S4

Patients characteristics	*C/C*, n=173	*G/C*, n=146	*G/G*, n=27	*P* value
Age, years	57.2±10.8	54.0±10.6	51.9±8.8	**0.006**
Sex				
Male	109 (63.0%)	100 (68.5%)	21 (77.8%)	0.253
Female	64 (37.0%)	46 (31.5%)	6 (22.2%)	
History of hepatic decompensation	18 (10.4%)	19 (13.0%)	7 (25.9%)	0.079
Varices	36 (20.8%)	45 (30.8%)	11 (40.7%)	**0.029**
Small	18 (10.4%)	26 (17.8%)	3 (11.1%)	**0.019**
Large	18 (10.4%)	19 (13.0%)	8 (29.6%)	
BL-CTP score, points	5±1	6±1	6±1	0.046
Stage A	161 (93.1%)	121 (82.9%)	22 (81.5%)	**0.012**
Stage B/C	12 (6.9%)	25 (17.1%)	5 (18.5%)	
BL-MELD score, points	8.4±2.4	9.4±3.2	9.2±2.5	**0.005**
BL-LSM[1], kPa	16.9 (11.8-26.6)	18.0 (11.7-30.0)	29.1 (21.6-38.5)	**0.005**
Evidence of CSPH[2]	76 (43.9%)	83 (56.8%)	22 (81.5%)	**<0.001**
BL-PLT, G x L^{-1}	145±66	133±63	116±65	0.061
BL-VWF[3], %	232 (167-307)	245 (183-329)	292 (213-379)	0.167
BL-VITRO[3]	1.65 (0.99-3.23)	1.94 (1.17-3.30)	2.74 (1.34-4.78)	**0.038**

[1] Information available in 310 patients.

[2] Defined by history of hepatic decompensation, presence of varices, or LSM ≥20kPa.

[3] Information available in 328 patients.

Table S4. Patient characteristics in the overall cohort (**cohort B**) and comparison between *PNPLA3 rs738409* genotypes.

Abbreviations:
BL baseline
CTP Child-Turcotte-Pugh score
MELD model for end-stage liver disease
CSPH clinically significant portal hypertension

PLT platelet count

VWF von Willebrand factor

VITRO von Willebrand factor antigen/platelet count ratio

Table S5

Patients characteristics	All, n=65	HVPG-decrease ≥10%, n=39	No HVPG-decrease ≥10%, n=26	*P* value
Age, years	54.6±9.4	55.9±8.9	52.8±10.0	0.198
Sex				
Male	44 (67.7%)	25 (64.1%)	19 (73.1%)	0.448
Female	1 (32.3%)	14 (35.9%)	7 (26.9%)	
BMI, kg x m^{-2}	25.6±4.6	25.5±4.2	25.7±5.3	0.899
≥25 kg x m^{-2}	33 (50.8%)	19 (48.7%)	14 (53.8%)	0.685
≥30 kg x m^{-2}	11 (16.9%)	6 (15.4%)	5 (19.2%)	0.685
Alcohol consumption				
Abstinent	55 (84.6%)	33 (84.6%)	22 (84.6%)	0.435
Non-abstinent but below threshold[1]	5 (7.7%)	4 (10.3%)	1 (3.8%)	
Above threshold[1]	5 (7.7%)	2 (5.1%)	3 (11.5%)	
PNPLA3 rs738409				
C/C	29 (44.6%)	16 (41.0%)	13 (50.0%)	0.761
C/G	27 (41.5%)	17 (43.6%)	10 (38.5%)	
G/G	9 (13.8%)	6 (15.4%)	3 (11.5%)	
G/C or *G/G*	36 (55.4%)	23 (59.0%)	13 (50.0%)	0.476
TM6SF2 rs58542926				
C/C	53 (81.5%)	32 (82.1%)	21 (80.8%)	1.000
C/T	12 (18.5%)	7 (17.9%)	5 (19.2%)	
MBOAT7 rs641738				
C/C	13 (20.0%)	9 (23.1%)	4 (15.4%)	0.710
C/T	39 (60.0%)	22 (56.4%)	17 (65.4%)	
T/T	13 (20.0%)	8 (20.5%)	5 (19.2%)	
HSD17B13 rs72613567				
T/TA	42 (64.6%)	27 (69.2%)	15 (57.7%)	0.224
T/TA	21 (32.3%)	10 (25.6%)	11 (42.3%)	
TA/TA	2 (3.1%)	2 (5.1%)	0 (0.0%)	
Allele score, points	1 (1-2)	1 (1-2)	2 (0-2)	0.647
History of hepatic decompensation	13 (20.0%)	7 (17.9%)	6 (23.1%)	0.613
Varices	31 (47.7%)	17 (43.6%)	14 (53.8%)	0.417
Small	15 (23.1%)	10 (25.6%)	5 (19.2%)	0.309
Large	16 (24.6%)	7 (17.9%)	9 (34.6%)	

BL-CTP score, points	6±1	5±1	6±1	0.080
Stage A	56 (86.1%)	35 (89.7%)	21 (80.8%)	0.465
Stage B/C	9 (13.8%)	4 (10.3%)	5 (19.2%)	
BL-MELD score, points	9.8±2.6	9.1±1.8	10.9±3.1	**0.012**
BL-HVPG, mmHg	16.1±4.4	15.7±4.0	16.6±4.9	0.438
10-15mmHg	29 (44.6%)	19 (48.7%)	10 (38.5%)	0.415
≥16mmHg	36 (55.4%)	20 (51.3%)	16 (61.5%)	
BL-LSM[2], kPa	27.4 (21.3-45.0)	27.0 (20.9-40.6)	28.6 (23.3-47.4)	0.360
BL-PLT, G x L^{-1}	101±47	111±48	87±41	**0.036**
BL-VWF[2], %	293 (218-366)	292 (191-350)	328 (233-385)	0.266
BL-VITRO[2]	3.30 (2.00-5.50)	2.84 (1.62-4.02)	4.63 (2.54-6.28)	**0.012**

[1] >30g/day and >20g/day for males and females, respectively[2].

[2] Information available in 63 patients.

Table S5. Baseline characteristics of **cohort A** patients with CSPH and comparison between patients with a HVPG-decrease ≥10%, or without.

Abbreviations:

BMI body mass index

CSPH clinically significant portal hypertension

CTP Child-Turcotte-Pugh score

HSD17B13 hydroxysteroid 17-beta dehydrogenase 13

LSM liver stiffness measurement

MBOAT7 membrane bound O-acyltransferase domain containing 7

MELD model for end-stage liver disease

PLT platelet count

PNPLA3 patatin-like phospholipase domain-containing protein 3

TM6SF2 transmembrane 6 superfamily member 2

VITRO von Willebrand factor antigen/platelet count ratio

VWF von Willebrand factor

Table S6

Patients characteristics	No hepatic decompensation during FU, n=323	Hepatic decompensation during FU, n=23[1]	*P* value
Age, years	55.7±10.6	51.4±9.3	0.063
Sex			
Male	214 (66.3%)	16 (69.6%)	0.745
Female	109 (33.7%)	7 (30.4%)	
PNPLA3 rs738409			
C/C	165 (51.1%)	8 (34.8%)	
G/C	135 (41.9%)	11 (47.8%)	0.121
G/G	23 (7.1%)	4 (17.4%)	
G/C or *G/G*	158 (48.9%)	15 (65.2%)	0.131
History of hepatic decompensation	29 (9.0%)	15 (65.2%)	**<0.001**
Varices	74 (22.9%)	18 (78.3%)	**<0.001**
Small	41 (12.7%)	6 (26.1%)	**<0.001**
Large	3 (10.2%)	12 (52.2%)	
BL-CTP score, points	5±1	7±1	**<0.001**
Stage A	295 (91.3%)	9 (39.1%)	**<0.001**
Stage B/C	28 (8.7%)	14 (60.9%)	
BL-MELD score, points	8.6±2.7	12.1±2.2	**<0.001**
BL-LSM[2], kPa	17.2 (11.8-27.0)	39.3 (28.0-59.1)	**<0.001**
Evidence of CSPH[3]	158 (48.9%)	23 (100%)	**<0.001**
BL-PLT, G x L^{-1}	141±65	87±43	**<0.001**
BL-VWF[4], %	233 (170-309)	371 (258-420)	**<0.001**
BL VITRO[4]	1.78 (1.06-3.16)	4.00 (2.34-7.00)	**<0.001**
BL-bilirubin, mg x dL^{-1}	0.72 (0.53-1.07)	1.24 (0.70-1.90)	**0.001**
BL-creatinine, mg x dL^{-1}	0.78 (0.69-0.91)	0.80 (0.64-0.91)	0.806
BL-albumin, g x L^{-1}	41.0±4.7	34.7±4.8	**<0.001**
BL-INR	1.16±0.26	1.44±0.25	**<0.001**
BL-AST, U x L^{-1}	68 (43-101)	66 (43-127)	0.653
BL-ALT, U x L^{-1}	69 (43-109)	49 (29-91)	0.125
BL-GGT, U x L^{-1}	94 (53-160)	90 (35-180)	0.760

[1] Nine patients (3.9%) showed development/worsening of ascites, 8 patients (2.3%) had development/worsening of HE, and 6 patients (1.7%) suffered from variceal bleeding as first decompensation event post-treatment.

[2] Information available in 310 patients.

[3] Defined by history of hepatic decompensation, presence of varices, or LSM ≥20kPa.

[4] Information available in 328 patients.

Table S6. Patient characteristics of the overall cohort (**cohort B**) and comparison between patients with hepatic decompensation during FU, or without.

Abbreviations:

ALT alanine aminotransferase

AST aspartate aminotransferase

BL baseline

CSPH clinically significant portal hypertension

CTP Child-Turcotte-Pugh score

GGT gamma-glutamyltransferase

FU follow-up

HE hepatic encephalopathy

INR international normalized ratio

LSM liver stiffness measurement

MELD model for end-stage liver disease

PLT platelet count

PNPLA3 patatin-like phospholipase domain-containing protein 3

VITRO von Willebrand Factor antigen/platelet count ratio

VWF von Willebrand factor

Table S7

	A			B		
	Hepatic decompensation			Transplant-free mortality		
	aHR	95%CI	*P value*	aHR	95%CI	*P value*
PNPLA3 rs738409 G-allele carriers, vs. non-carriers	1.377	0.488-3.886	0.546	1.089	0.301-3.937	0.897
History of decompensation	7.342	2.573-20.948	**<0.001**	6.301	1.364-29.113	**0.018**
BL-MELD score, per point	1.248	1.066-1.462	**0.006**	0.871	0.615-1.234	0.437
BL-albumin, per g/L	0.890	0.804-0.985	**0.024**	0.871	0.743-1.021	0.088

Table S7. Cox regression analyses on the influence of the *PNPLA3 rs738409 G*-allele **(A)** on hepatic decompensation and **(B)** on transplant-free mortality in the overall cohort (**cohort B**).

Abbreviations:
CSPH clinically significant portal hypertension
MELD model for end-stage liver disease
PNPLA3 patatin-like phospholipase domain-containing protein 3

Figure S1

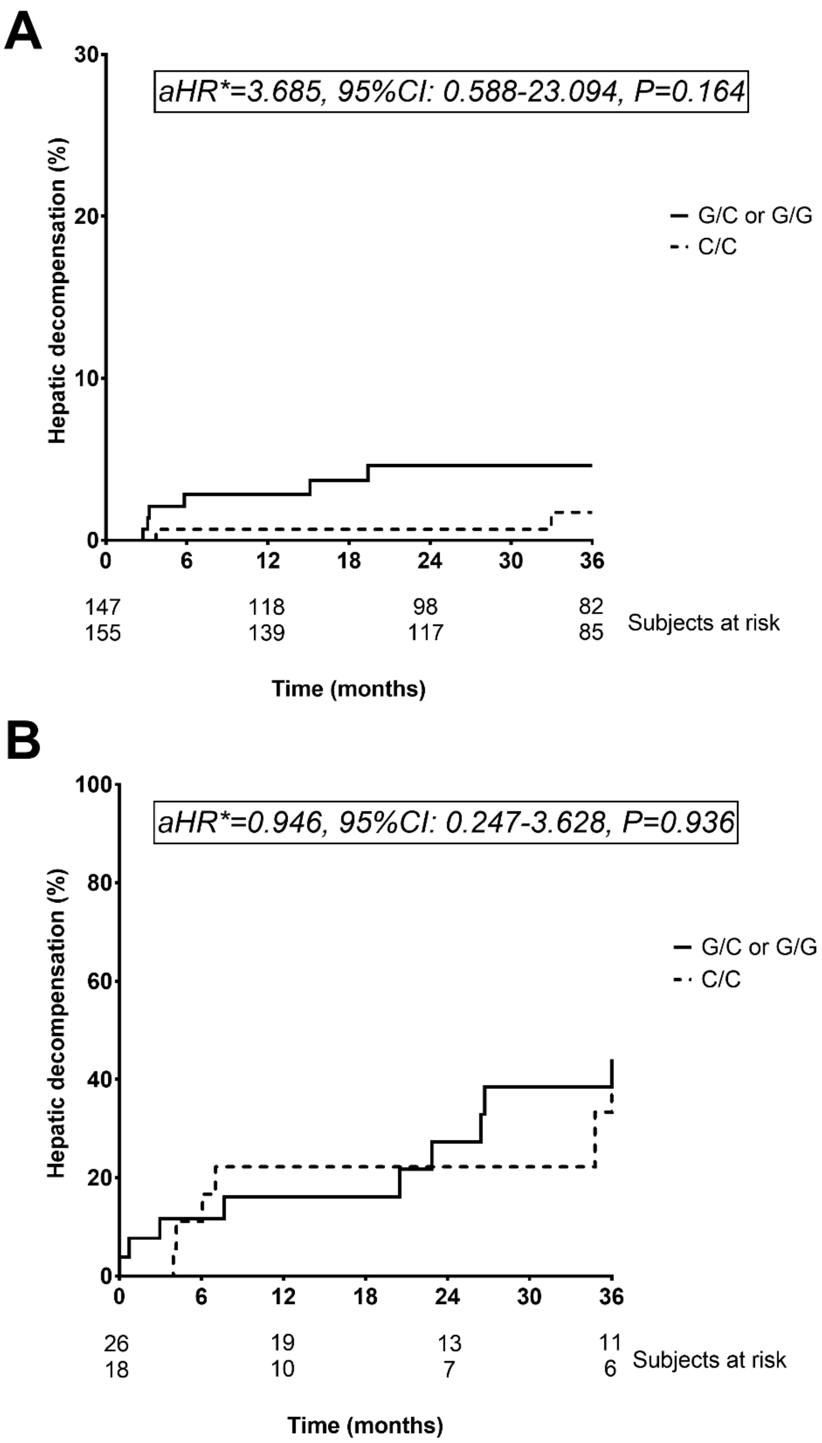

Figure S1. Kaplan-Meier analyses on **(A)** first hepatic decompensation in patients with cACLD and **(B)** further decompensation in dACLD patients of the overall cohort (**cohort B**), comparing carriers and non-carriers of *PNPLA3 rs738409 G*-allele.

Adjusted hazard ratio (aHR) was calculated using Cox regression analysis adjusting for baseline MELD score and baseline albumin level.

Abbreviations:

aHR adjusted hazard ratio

cACLD compensated advanced chronic liver disease

dACLD decompensated advanced chronic liver disease

PNPLA3 patatin-like phospholipase domain-containing protein 3

Figure S2

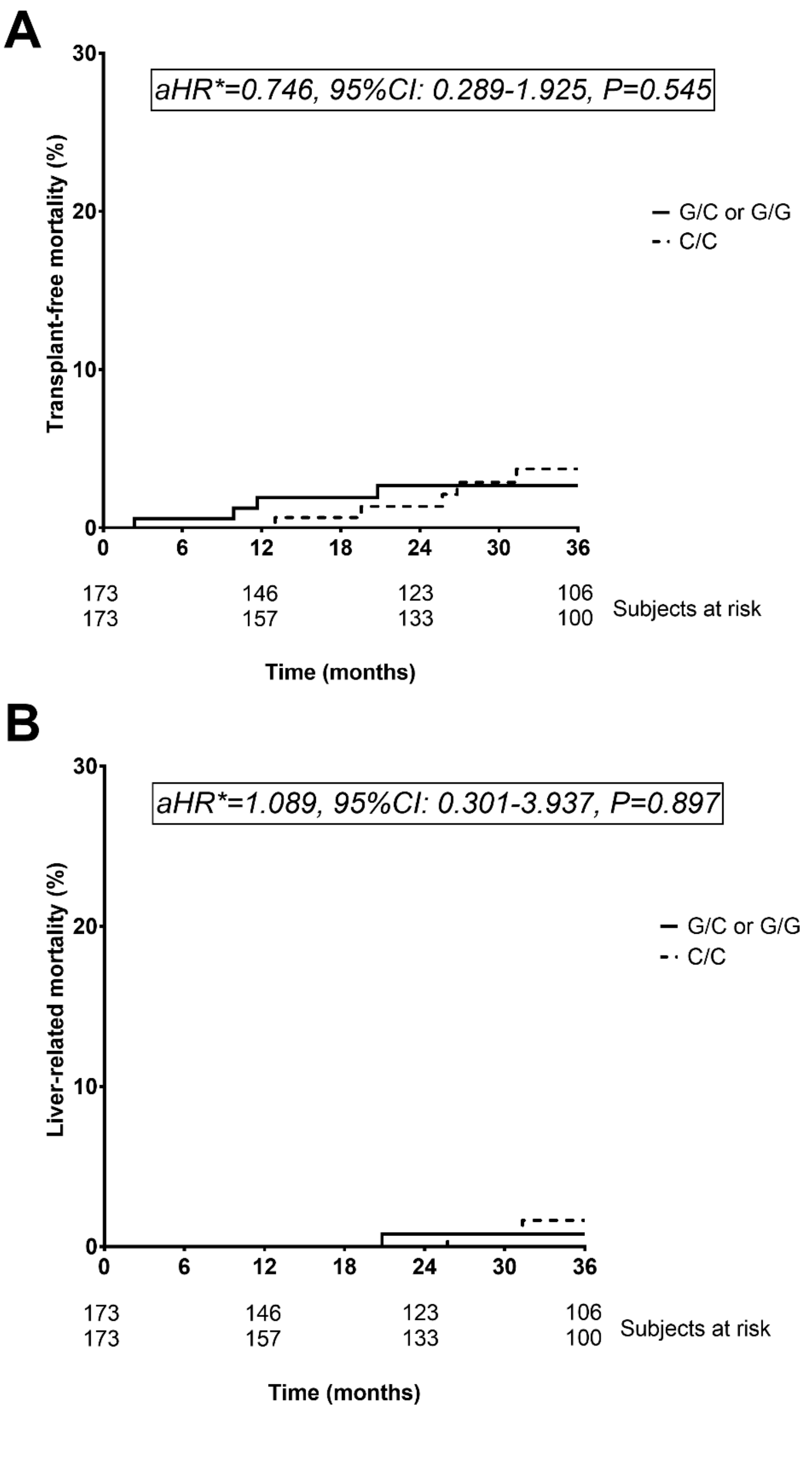

Figure S2. Kaplan-Meier analyses on **(A)** transplant-free mortality and **(B)** liver-related mortality in the overall cohort (**cohort B**), comparing carriers and non-carriers of *PNPLA3 rs738409 G*-allele. Adjusted hazard ratio (aHR) was calculated using Cox

regression analysis adjusting for history of hepatic decompensation, baseline MELD score and baseline albumin level.

Abbreviations:

aHR adjusted hazard ratio

PNPLA3 patatin-like phospholipase domain-containing protein 3

Figure S3

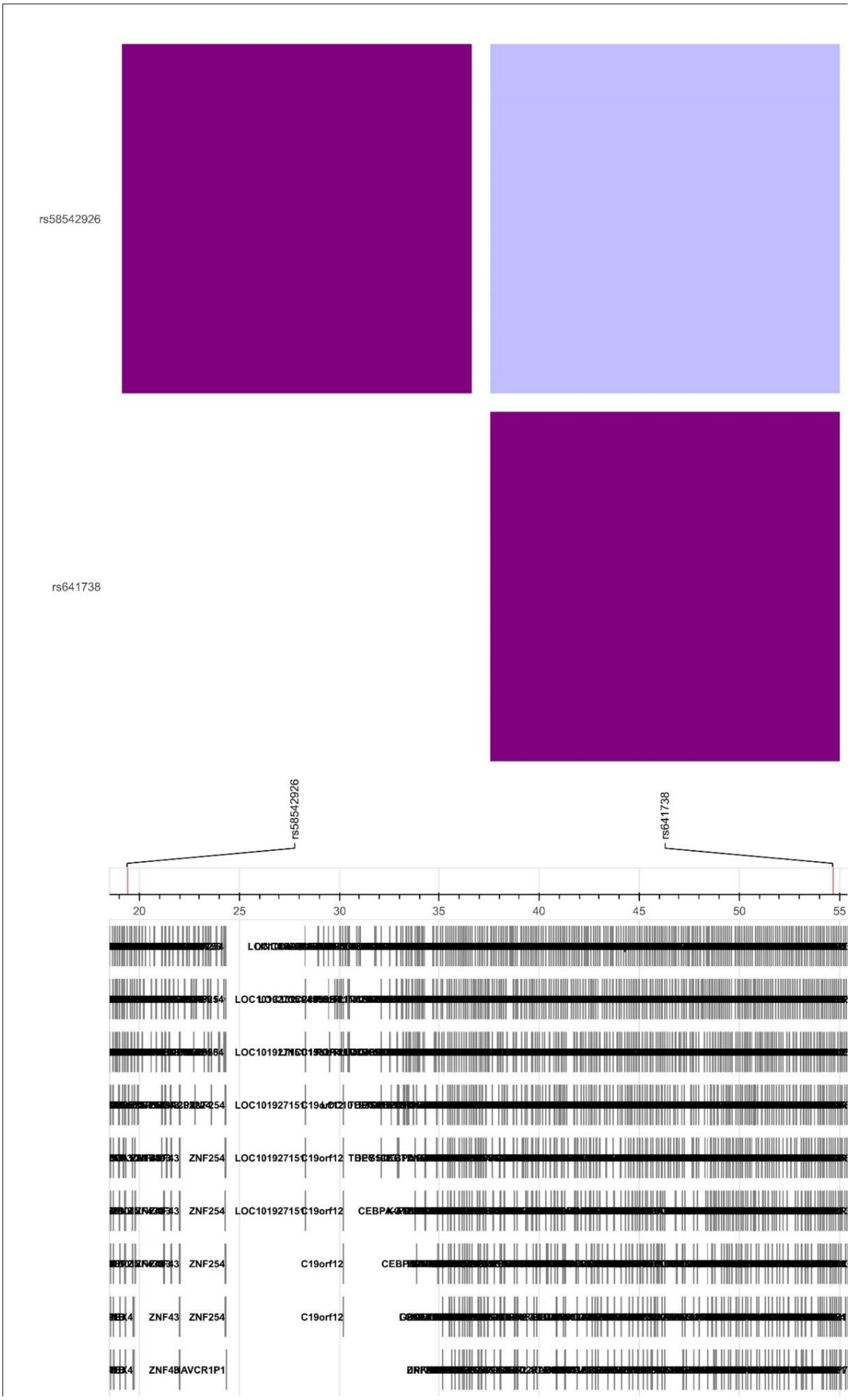

rs58542926
rs641738
rs58542926
rs641738
20
25
30
35
40
45
50
55
ZNF254
LOC101927151
C19orf12
CEBPA
ZNF43
VCR1P1

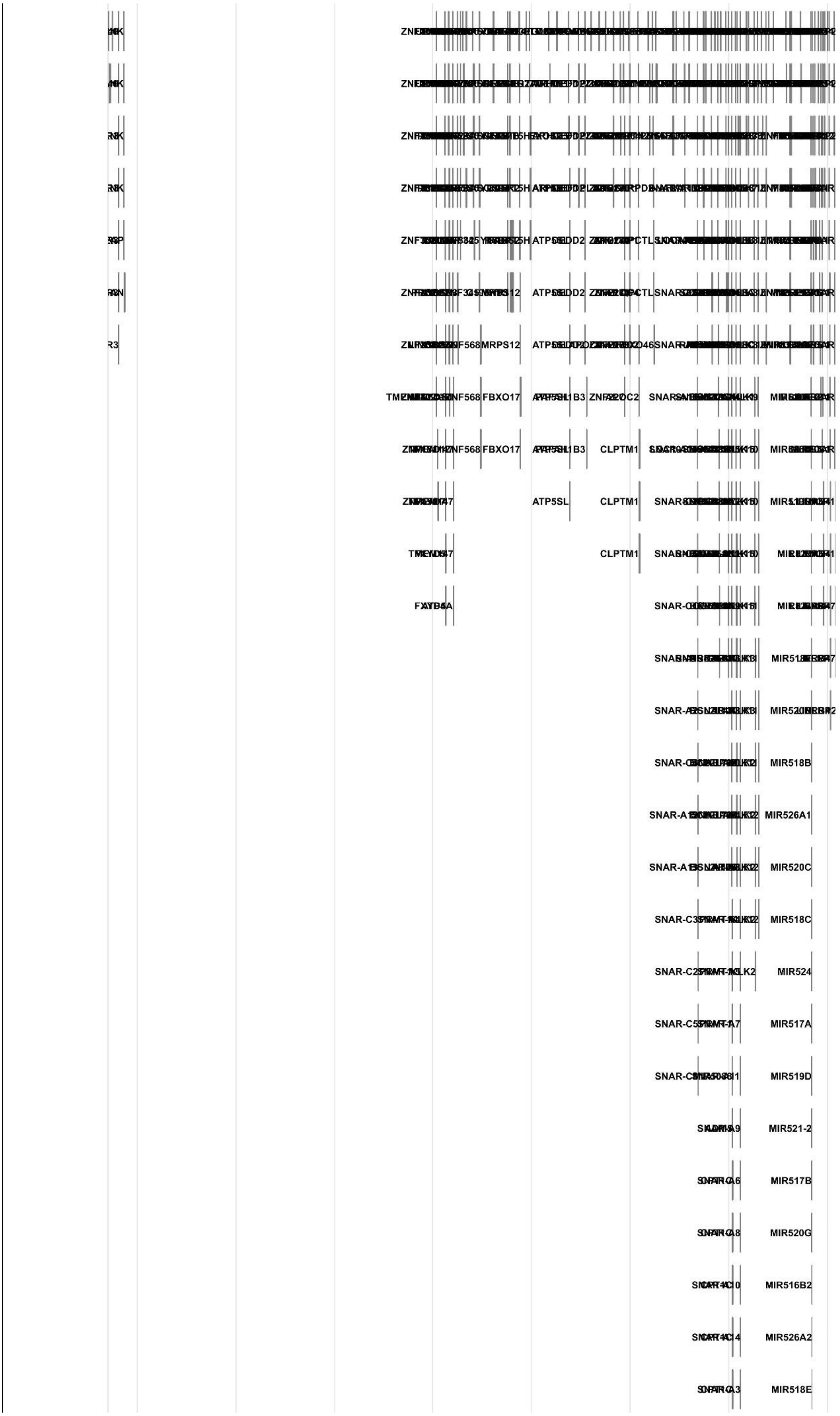
ZNF568
MRPS12
FBXO17
ATP5SL
CLPTM1
MIR518B
MIR526A1
MIR520C
MIR518C
MIR524
MIR517A
MIR519D
MIR521-2
MIR517B
MIR520G
MIR516B2
MIR526A2
MIR518E

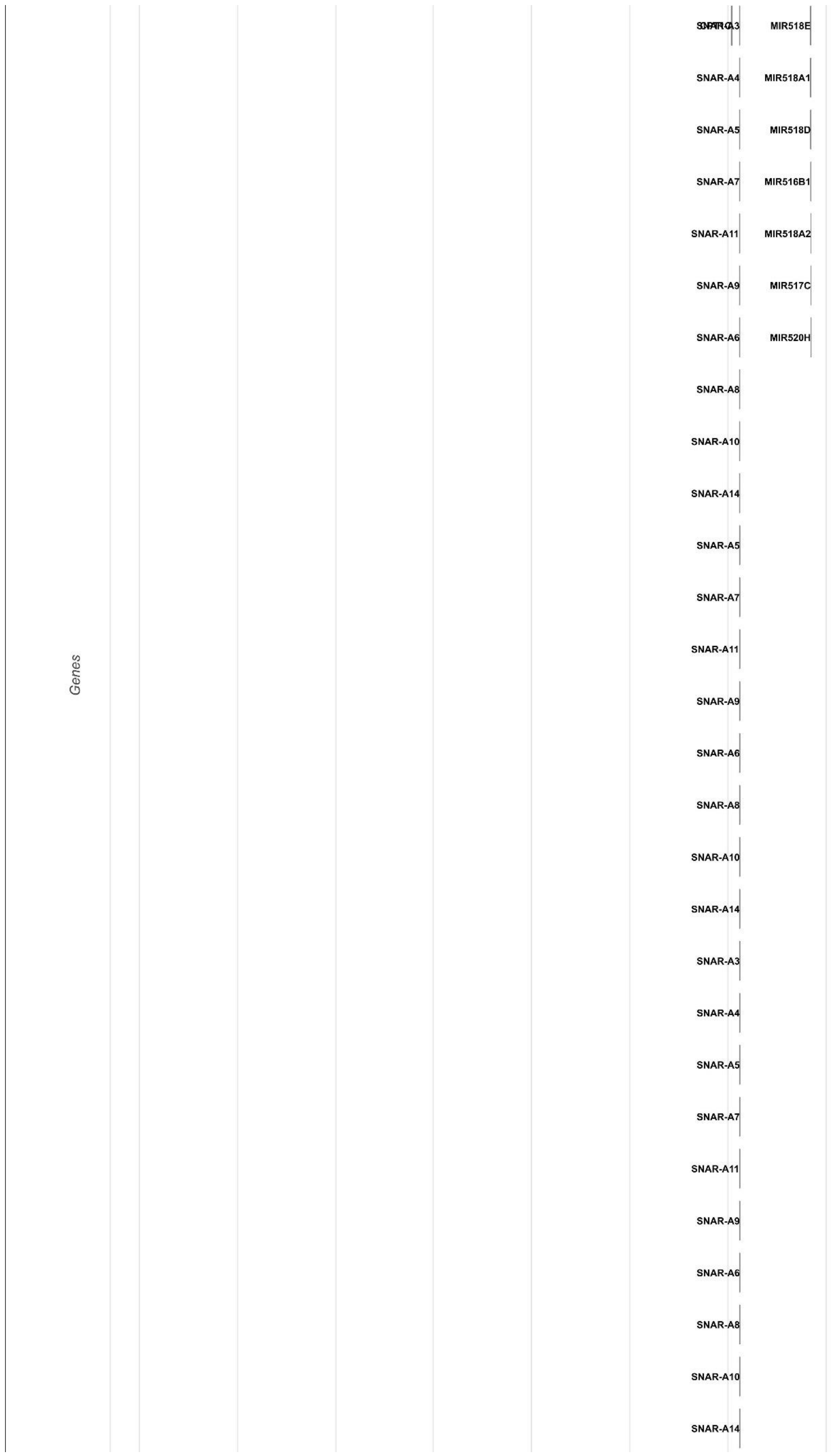
Genes
MIR518E
SNAR-A4
MIR518A1
SNAR-A5
MIR518D
SNAR-A7
MIR516B1
SNAR-A11
MIR518A2
SNAR-A9
MIR517C
SNAR-A6
MIR520H
SNAR-A8
SNAR-A10
SNAR-A14
SNAR-A5
SNAR-A7
SNAR-A11
SNAR-A9
SNAR-A6
SNAR-A8
SNAR-A10
SNAR-A14
SNAR-A3
SNAR-A4
SNAR-A5
SNAR-A7
SNAR-A11
SNAR-A9
SNAR-A6
SNAR-A8
SNAR-A10
SNAR-A14

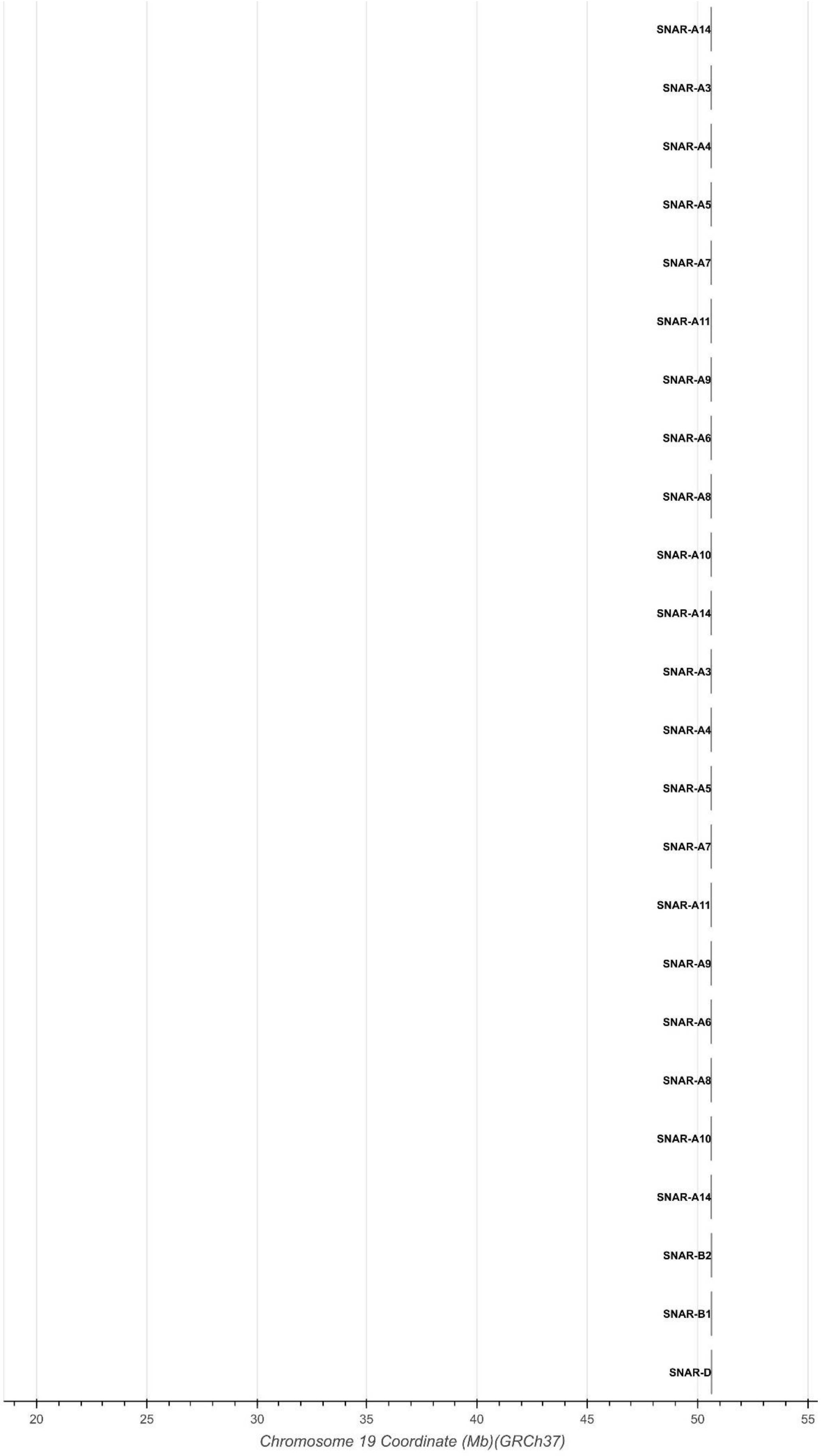

SNAR-A14
SNAR-A3
SNAR-A4
SNAR-A5
SNAR-A7
SNAR-A11
SNAR-A9
SNAR-A6
SNAR-A8
SNAR-A10
SNAR-A14
SNAR-A3
SNAR-A4
SNAR-A5
SNAR-A7
SNAR-A11
SNAR-A9
SNAR-A6
SNAR-A8
SNAR-A10
SNAR-A14
SNAR-B2
SNAR-B1
SNAR-D
20
25
30
35
40
45
50
55
Chromosome 19 Coordinate (Mb)(GRCh37)

Figure S3. Heatmap matrix of linkage disequilibrium statistics for TM6SF2 *rs58542926* and MBOAT7 *rs641738* based on in the ‘Toscani in Italia’ (TSI) cohort using LDmatrix (National Cancer Institute, https://ldlink.nci.nih.gov/?tab=ldmatrix). There was no evidence of a linkage disequilibrium between *TM6SF2 rs58542926 and MBOAT7 rs641738* in this cohort (D'=0.0126, R^2=0.0001, X^2=0.0117, *P*=0.9138).

References used in this supplementary material

1. Rodriguez, S.; Gaunt, T.R.; Day, I.N. Hardy-Weinberg equilibrium testing of biological ascertainment for Mendelian randomization studies. *American journal of epidemiology* **2009**, *169*, 505-514, doi:10.1093/aje/kwn359.
2. EASL-EASD-EASO Clinical Practice Guidelines for the management of non-alcoholic fatty liver disease. *Journal of hepatology* **2016**, *64*, 1388-1402, doi:10.1016/j.jhep.2015.11.004.
3. Alberti, K.G.; Zimmet, P.Z. Definition, diagnosis and classification of diabetes mellitus and its complications. Part 1: diagnosis and classification of diabetes mellitus provisional report of a WHO consultation. *Diabet. Med.* **1998**, *15*, 539-553, doi:10.1002/(SICI)1096-9136(199807)15:7<539::AID-DIA668>3.0.CO;2-S.
